# Nanomaterial‐assisted oncolytic bacteria in solid tumor diagnosis and therapeutics

**DOI:** 10.1002/btm2.10672

**Published:** 2024-04-17

**Authors:** Xiangdi Zeng, Qi Chen, Tingtao Chen

**Affiliations:** ^1^ Department of Obstetrics and Gynecology The Second Affiliated Hospital, Jiangxi Medical College, Nanchang University Nanchang Jiangxi China; ^2^ The First Clinical Medical College, Jiangxi Medical College, Nanchang University Nanchang Jiangxi China; ^3^ National Engineering Research Center for Bioengineering Drugs and the Technologies Institute of Translational Medicine, Jiangxi Medical College, Nanchang University Nanchang Jiangxi China; ^4^ School of Pharmacy Jiangxi Medical College, Nanchang University Nanchang Jiangxi China

**Keywords:** nanomaterial, oncolytic bacteria, tumor imaging, tumor therapy

## Abstract

Cancer presents a formidable challenge in modern medicine due to the intratumoral heterogeneity and the dynamic microenvironmental niche. Natural or genetically engineered oncolytic bacteria have always been hailed by scientists for their intrinsic tumor‐targeting and oncolytic capacities. However, the immunogenicity and low toxicity inevitably constrain their application in clinical practice. When nanomaterials, characterized by distinctive physicochemical properties, are integrated with oncolytic bacteria, they achieve mutually complementary advantages and construct efficient and safe nanobiohybrids. In this review, we initially analyze the merits and drawbacks of conventional tumor therapeutic approaches, followed by a detailed examination of the precise oncolysis mechanisms employed by oncolytic bacteria. Subsequently, we focus on harnessing nanomaterial‐assisted oncolytic bacteria (NAOB) to augment the effectiveness of tumor therapy and utilizing them as nanotheranostic agents for imaging‐guided tumor treatment. Finally, by summarizing and analyzing the current deficiencies of NAOB, this review provides some innovative directions for developing nanobiohybrids, intending to infuse novel research concepts into the realm of solid tumor therapy.

**Translational Impact Statement**

While oncolytic bacterial cancer therapy has been recognized as a promising therapeutic approach, concerns persist regarding its low pathogenicity, immunogenicity, and controllability. Nanomaterials represent a novel class of auxiliary substances, characterized by their small size, multifaceted properties, and favorable biocompatibility. Utilizing artificial methods to modify oncolytic bacteria with nanomaterials is pivotal in overcoming the inherent limitations of bacterial therapy and achieving complementarity of dual strengths.

## 1 INTRODUCTION

Oncolytic bacteria are genetically engineered or naturally occurring bacterial species with the remarkable ability to selectively target, colonize, and ultimately eradicate cancer cells. These multi‐mechanistic therapeutic agents can suppress tumors by directly inducing programmed and unprogrammed tumor cell death, disrupting tumor blood vessels, and activating innate or adaptive immune responses.^1^, ^2^, ^3^, ^4^, ^5^ Numerous types and strains of bacteria, such as *Salmonella*, *Escherichia coli* (*E. coli*), *Clostridium*, and *Listeria*, have been tested in preclinical and clinical trials and classified as oncolytic bacteria.^6^, ^7^, ^8^, ^9^ Notably, these promising candidates for advanced cancer treatment strategies show preferential colonization of hypoxic regions within tumors,^10^ excellent intratumoral penetration,^11^ and the ability to deliver drugs via convenient genetic manipulation.^12^ Despite exhibiting unique characteristics, oncolytic bacteria often pose inevitable side effects and dose‐dependent toxicity due to their high pathogenicity and immunogenicity, even in instances using live attenuated strains.^13^ Additionally, the limitations imposed by the single route of administration and the body's inherent clearance mechanisms lead to insufficient intratumoral colonization and accumulation.^14^, ^15^ Low clinical efficacy severely restricted the advancement and practical application of oncolytic bacteria, necessitating the urgent development of strategies that enhance effectiveness while minimizing risks.

Nanomaterials are materials with at least one dimension within the 1–100 nanometer scale, exhibiting exceptional optical, electrical, magnetic, and thermodynamic properties.^16^ As scaled down to the nanoscale, four effects differentiate nanomaterials from macroscopic materials: small size, quantum, surface, and retention effects.^17^, ^18^, ^19^ These effects enable nanomaterials to assume distinctive benefits in tumor diagnosis and treatment, such as ideal biocompatibility, drug‐targeted delivery, controlled release, and multimode bioimaging.^20^, ^21^ Integrating customized functional materials with oncolytic bacteria holds immense potential and practical value in tumor therapy. As targeted carriers, nanomaterials surface‐modified with different molecules like chemotherapeutic drugs and genes present an ideal solution for minimizing damage to normal cells and mitigating undesired toxicity.^22^, ^23^ They can not only improve bacterial adhesion and colonization at tumor sites but also provide precise control over drug solubility, stability, and release in the intracellular and extracellular environment.^24^, ^25^, ^26^ As therapeutic agents, multifunctional nanomaterials bestow oncolytic bacteria with characteristics that surpass their inherent capabilities. The physicochemical properties of nanomaterials allow oncolytic bacterial therapy to synergize therapeutic effects with photothermal therapy (PTT), photodynamic therapy (PDT), chemotherapy, radiotherapy, immunotherapy, and other modalities.^27^, ^28^, ^29^, ^30^, ^31^ These nanomaterials also serve as imaging probes, generating specific signals for tumor therapy visualization.^32^ Nanomaterial‐assisted oncolytic bacteria (NAOB) further broaden the means and modes of oncolytic bacterial therapy, leading to precision tumor therapy.

This review comprehensively summarizes the various applications of NAOB in solid tumor diagnosis and treatment. Specifically, we focus on leveraging them to augment the tumor's therapeutic efficacy and act as nanotheranostic agents for visualizing treatment pathways. Besides, to fully exploit the potential of NAOB in clinical practice, we undertake a rigorous assessment of the current research in this field and provide recommendations for further investigations. Overall, this review provides an essential reference for researchers and clinicians to expand the indications for bacterial‐mediated tumor therapy and develop innovative and effective treatment strategies for solid tumors.

## 2 ASSESSMENT OF TUMOR THERAPY: MERITS AND DEMERITS OF CONVENTIONAL TREATMENT METHODS

Cancer is a severe global public health problem with extremely high morbidity and mortality. According to the American Cancer Society, in 2024, there will be an estimated 2,001,140 new cases and 611,720 deaths from cancer in the United States.^33^ Tumor development, characterized by aberrant cell differentiation and hyper‐proliferation, is a complex process.^34^ Composed of these aberrant cells, the tumor microenvironment (TME) is hypoxic, acidic, and immunosuppressive, which makes solid tumors challenging to treat. The hypoxic TME triggers anaerobic glycolysis in tumor cells and stimulates the production of neovascularization factors, like vascular endothelial growth factor (VEGF).^35^ Anaerobic glycolysis exacerbates excess lactic acid production and activates specific proteases, which may be responsible for turning off some chemotherapy drugs.^36^ VEGF induces vascular hyper‐permeability and promotes abnormal neovascularization,^37^ contributing to generating the immunosuppressive TME.^38^, ^39^ Encouragingly, these current challenges in cancer treatment have vigorously promoted the continuous development of novel therapeutic approaches.

Surgery, radiotherapy, and chemotherapy are orthodox treatment methods for solid tumors, and each has unique virtues and drawbacks. Surgical extirpation is the preferred choice for most patients with early or intermediate‐stage solid tumors, which is highly effective and easy to perform. Tumors and corresponding organs can be removed directly without considering cell proliferation or treatment sensitivity. However, surgery impairs the immune system and may not guarantee complete cancer cell eradication, bringing about recurrence and metastasis of some malignant tumors.^40^ A recent study demonstrated that neutrophils were activated and subsequently underwent NETosis in the TME and nearby surgical wound, which may be a vital postoperative risk factor for postoperative tumor relapse and metastasis.^41^

While exerting therapeutic effects, radiotherapy can also induce DNA damage by causing double‐strand breaks, leading to cell apoptosis or necrosis.^42^ Additionally, radiation contributes to genetic mutations, mainly characterized by small fragment deletions closely associated with tumor radiotherapy resistance.^43^ Another feature of radiotherapy is that the therapeutic effect often depends on cell susceptibility to radiation. Scientists showed that the imbalanced cytokinesis of normal esophageal cells might make it difficult for radiation to damage all oesophageal squamous cell carcinoma cells.^44^ With the higher fraction of dividing daughter cells, an aberrant rise in cell proliferation rates and squeezing of surrounding normal esophageal epitheliums resulted in tumor recurrence. However, high‐precision radiotherapy is capable of reducing local recurrence,^45^ downsizing the primary tumor,^46^ and improving overall survival. For patients who cannot undergo surgery, radiotherapy is considered a life‐prolonging alternative.^47^ The above traits make palliative and curative radiotherapy recommended for nasopharyngeal carcinoma and prostate cancer.^48^, ^49^

Different from surgery and radiotherapy, chemotherapy is usually a systemic therapy. Chemotherapeutic agents damage tumor cells by interfering with cell division, dysregulating cellular metabolism, and inhibiting nucleic acid or protein biosynthesis.^50^ Multidrug resistance is a significant factor in chemotherapy failure, and it may involve protein–protein interactions, oxidative stress, and genomic mutations. The WNT16B released by fibroblasts after chemotherapy damage can weaken the cytotoxicity of chemotherapy drugs in vivo and promote tumor cell survival.^51^ High‐level reactive oxygen species (ROS) are critical in maintaining cellular redox balance. However, the interaction between the inhibitor of apoptosis‐stimulating protein of p53 (iASPP) and the antioxidant core factor Nrf2 could suppress their production. The iASPP/Nrf2/ROS signaling pathway may be pivotal in renal carcinoma resistance to 5‐fluorouracil (5‐FU).^52^ During neoadjuvant chemotherapy, tumor cells changed their genome and phenotypic evolution in triple‐negative breast cancer.^53^ The experimental results indicate that resistant genotypes are pre‐existing and adaptively selected by cancer cells. With the increasing emphasis on individualization and low side effects in cancer therapy, molecular‐targeted therapy has emerged. Although the efficacy of molecular‐targeted therapy varies widely among individuals, it is possible to achieve long‐term disease control by combining it with selective drug combinations for different tumor resistance mechanisms.^54^

Despite the plethora of available tumor treatment methods, it has become increasingly apparent in clinical practice that each approach needs to be revised. In 2019, an article in *Nature* reported that an engineered *E. coli* strain could induce sustained tumor regression.^31^ This study first substantiated an abscopal effect caused by oncolytic bacteria, showcasing their ability to generate potent, tumor‐specific adaptive immune responses with systemic efficacy in clearing distant tumor lesions. Subsequently, mounting evidence indicates the substantial promise of oncolytic bacteria in tumor therapy.

## 3 ONCOLYTIC BACTERIAL THERAPY: CURRENT STATUS AND PROGRESS

### 3.1 The history of oncolytic bacterial therapy

The origin of oncolytic bacterial therapy dates back to the late nineteenth century when William Coley creatively injected a combination of heat‐killed *Streptococcus pyogenes* and *Serratia marcescens* (known as Coley's toxin) into patients with osteosarcoma.^55^ However, the high degree of individual variability, poor reproducibility, and unknown mechanisms severely hampered this approach as a routine oncology treatment. Progress in this field met a turning point in 2012 when scientists, such as Karbach, reported that the mechanism of Coley's toxin was to reawaken the host's immune system to suppress tumor cells.^56^ This new finding renewed researchers' interest in Coley's toxin, with Teoh following Coley's work and validating that heat‐inactivated *Clostridium sporogenes* reduced the proliferation of CT26 and HCT116 colorectal cancer cells to 20% and 26.2%, respectively.^57^ These works suggest that bacterial therapeutics is a promising area for further investigation by researchers as a novel approach to dealing with solid tumors.

### 3.2 Multifaceted oncolytic mechanisms

**FIGURE 1:**
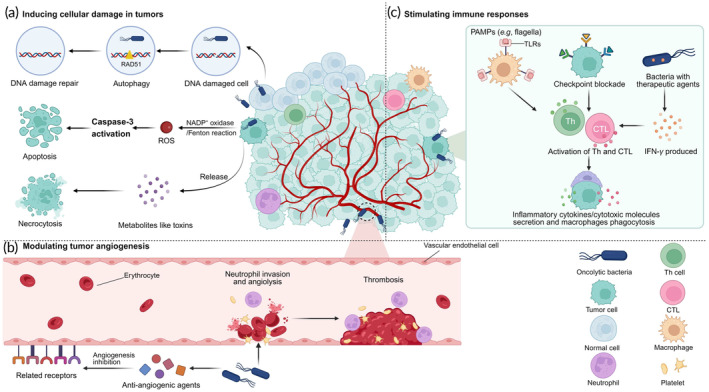
A comprehensive overview of the mechanisms underlying tumor eradication through oncolytic bacteria.

Oncolytic bacteria can impact tumor cells directly or indirectly, and the three main potential mechanisms are as follows: (1) induction of programmed cell death and unprogrammed necrosis in tumor cells, (2) modulation of tumor angiogenesis through vascular destruction or inhibition, and (3) activation of the immune system to suppress tumorigenesis (Figure 1).

The extensively investigated pathways of tumor cell death directly triggered by oncolytic bacteria encompass programmed cell death, including autophagy and apoptosis, as well as unprogrammed necrosis. Autophagy is a catabolic process widespread in eukaryotes and essentially a lysosome‐dependent protein degradation pathway.^58^ Lucas et al. observed high mRNA expression levels of specific autophagy‐related genes when the colibactin‐producing *E. coli* (CoPEC) strain colonized the intestinal mucosa. It suggested that CoPEC infection triggered autophagy in colonic mucosal epithelial cells, potentially preventing DNA double‐strand breaks in healthy cells and the occurrence of colorectal cancer.^1^ The caspase family of cysteine proteases directly leads to apoptotic and is central to the network of apoptotic mechanisms. Research has shown that engineered *Salmonella Typhimurium* (*S. Typhimurium*) SL7207 transfected with a luminescent gene cluster induced caspase‐dependent apoptosis in HepG2 hepatocellular carcinoma cells by activating Caspase‐3.^2^ Intracellular pro‐apoptotic signaling molecules include oxidative stress (ROS, glutathione, etc.), cytochrome C, calcium ions (Ca^2+^), and endoplasmic reticulum stress. Among these, ROS are crucial to caspase‐independent apoptosis.^59^ *Lactobacillus* can enhance multifarious ROS‐dependent apoptosis‐inducing signals,^60^ while *Listeria monocytogenes* can increase intracellular levels of Ca^2+^ and ROS by activating nicotinamide adenine dinucleotide phosphate (NADP^+^) oxidase.^61^ Both of these mechanisms result in high levels of ROS production and directly induce apoptosis of tumor cells through intracellular oxidative damage. Utilizing oncolytic bacteria to produce cytotoxic proteins at the tumor site enables targeted destruction of non‐programmed cancer cell necrosis. Alpha‐toxin secreted by *Clostridium novyi‐*NT (*C. novyi*‐NT),^62^ the Exotoxin T produced by *Pseudomonas aeruginosa*,^63^ and the pore‐forming toxin produced by *Clostridium perfringens* ^64^ are among the various toxins that can exert anticancer effects by causing unprogrammed cell necrosis.

Combined therapy with oncolytic bacteria and angiogenesis inhibition strategies primarily focus on inhibiting neoangiogenesis or destroying existing tumor vasculature. Infections with *S. Typhimurium*, Zhao et al. observed a significant increase in local tumor neutrophil infiltration, hemoglobin content, and vascular endothelial cell damage.^65^ Simultaneously, the release of vasodilator inflammatory factors further exacerbated the formation of hemorrhagic inflammation, prompting blood outflow and the construction of thrombi. Qin and colleagues conducted a comparable study and found that infection with *E. coli* MG1655, expressing cytolysin A (ClyA), resulted in hemoconcentration at tumor sites with a substantial increase in hemoglobin levels. Eventually, it facilitated thrombosis, preventing tumor growth by disrupting the nutrient supply.^4^ A principal target of current anti‐angiogenic agents is the vascular endothelium cells (VECs), a pivotal participant in angiogenesis. Tumstatin and HM‐3 serve as two distinct tumor‐specific angiogenesis inhibitors. Tumstatin suppresses the proliferation of VECs by downregulating VEGF‐A, while HM‐3 inhibits the migration of VECs by binding to integrin *αvβ*3. Two independent studies have shown a noticeable decrease in the expression of platelet endothelial cell adhesion molecule‐1 (CD31, a marker of vascular endothelial differentiation) when utilizing *S. Typhimurium* VNP20009 in combination with Tumstatin and HM‐3. The investigation suggests the competence of oncolytic bacteria in effectively targeting tumor vessels and impeding the angiogenesis process.^66^, ^67^

The innate immune system employs pattern‐recognition receptors (PRRs) to recognize pathogen‐associated molecular patterns (PAMPs) in oncolytic bacteria, with Toll‐like receptors (TLRs) playing a crucial role in this initial recognition.^68^ *Salmonella* flagella can activate TLR5 in antigen‐presenting cells (APCs like macrophages).^69^ TLR5 further initiates the activation of the Nuclear factor kappa‐B (NF‐*κ*B) pathway, which promotes the enormous production of interferon (IFN), interleukin‐17 (IL‐17), and macrophage chemokines.^70^ Simultaneously, major histocompatibility complex class II molecules situated on APCs engage with the tyrosine kinase Btk through the co‐stimulatory molecule cluster of differentiation (CD) 40, facilitating the synthesis of tumor necrosis factor (TNF).^71^ Both pathways elicit robust responses from CD4^+^ helper T (Th) cells and CD8^+^ cytotoxic T cells (CTLs), ultimately leading to the potent antitumor effect through the secretion of inflammatory cytokines and cytotoxic molecules.^72^, ^73^ In concert with TNF signaling, lipopolysaccharide (LPS) activates downstream TLR4/NF‐*κ*B signaling pathway, augmenting intestinal B cell survival and proliferation.^74^ TLR2 recognizes lipoprotein,^75^ and TLR9 identifies bacterial DNA containing cytosine‐phosphate‐guanine (CpG) dideoxynucleotides.^76^ The rapid progress in synthetic biology has facilitated the innovative development of genetically engineered bacteria capable of producing and expressing various immunotherapeutics. IFN‐*γ* is a widely recognized cytokine immunomodulator known for its antitumor effects and stimulation of immune cells.^77^ In a recent study, researchers conducted attenuated *S. Typhimurium* with the ability to synthesize IL‐18, a factor known for inducing IFN‐*γ* production and immune cell activation.^78^ The stimulator of interferon genes (STING) is a cell‐intrinsic metabolic checkpoint in innate immunity.^79^ Daniel et al. genetically modified a strain of *E. coli* called SYNB1891 to express STING‐agonist cyclic diAMP. Upon intratumoral injection of SYNB1891 into murine tumors, a substantial upregulation of IFN‐*γ* was observed.^80^ Another fundamental mode of oncolytic bacteria‐mediated immunotherapy is the reactivation of the TME. Engineered *E. coli*, designed to deliver immune checkpoint inhibitors targeting programmed cell death protein‐ligand 1 (PD‐L1) and cytotoxic T‐lymphocyte‐associated protein‐4 (CTLA‐4), recruits and activates Th cells while reducing the proportion of regulatory T (Treg) cells.^81^ The injection of *E. coli* outer membrane vesicles (OMVs) as exogenous antigens into tumors similarly induced a durable therapeutic response mediated by the adaptive immune system.^82^

### 3.3 Advantages and disadvantages

Natural oncolytic bacteria can penetrate the peripheral bloodstream, accumulate and proliferate within hypoxic regions, and present the tumor‐suppressive effect.^10^ In contrast to inactivated bacteria, live bacteria have the fabulous talent to target tumors through intratumoral colonization. For example, *C. novyi* localized precisely in glioblastomas, whereas no colonization was observed in normal brain tissues.^83^ Bacterial motility can overcome the diffusion limitation of chemotherapeutic drugs, allowing them to penetrate tumor tissue and treat drug‐resistant areas away from the vasculature.^84^ Motility also influences the degree of colonization and spatial distribution of oncolytic bacteria.^85^ Unlike live cell‐dependent oncolytic viruses, oncolytic bacteria are safer and easier to control with medication.^86^ Despite numerous studies reporting the remarkable efficacy of oncolytic bacteria against tumors, their application as anticancer agents can be challenging. First, some strains remain toxic even at therapeutic doses, while reducing the dosage can compromise treatment efficacy.^14^, ^87^ Additionally, probiotic bacteria, like *Lactobacillus*, may be associated with potential side effects such as systemic infections, adverse metabolic activities, and excessive immune stimulation in susceptible individuals.^88^ Engineered bacteria maintain wild bacteria's kinetic activity and reduce toxicity. Within the appropriate dosage range, engineered bacteria are harmless to the organism and target and colonize tumors more precisely. Nonetheless, engineered bacteria exhibit inherent instability, and the potential for genetic mutations over time introduces the risk of functional escape.^89^, ^90^ Therefore, utilizing oncolytic bacterial therapy as a monotherapy cannot provide an efficient and safe approach to antitumor treatment.

## 4 ADVANCING SOLID TUMOR THERAPEUTICS: APPLICATIONS OF NANOMATERIAL‐ASSISTED ONCOLYTIC BACTERIA

**FIGURE 2:**
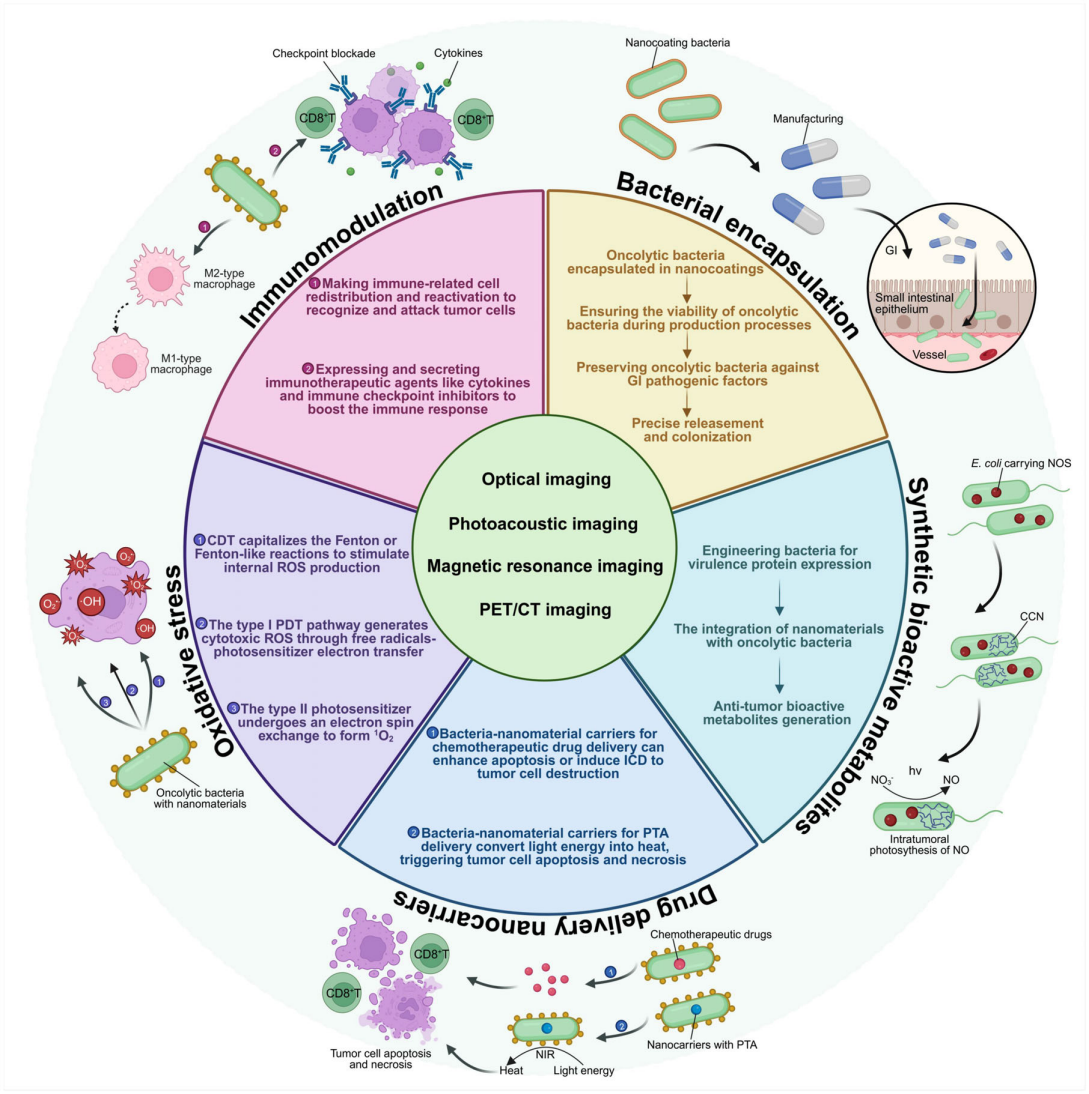
Conceptual diagram of nanomaterial‐assisted oncolytic bacteria used in various imaging‐guided solid tumor treatments.

The first section focuses on boosting therapeutic effectiveness through nanomaterial‐assisted strategies, which involve encapsulating oncolytic bacteria during tumor‐targeted transportation, collaboratively synthesizing antitumor agents, and modulating the TME to trigger immune responses. In the second section, we illustrate and list some representative bacterial‐nanotheranostic agents that integrate tumor imaging and treatment (Table 1). Generally, our review provides an analysis of the specific applications of NAOB in tumor treatment and visualization while offering insights into the bright prospects of this approach (Figure 2).

**TABLE 1:** Summary of representative nanomaterial‐assisted oncolytic bacteria utilized as nanotheranostic agents for tumor imaging and therapy.

Category	Bacteria	Nanomaterial	Imaging modality	Application	Ref.
Optical/Photoacoustic imaging‐guided nanotheranostic agent	Cyanobacteria	Ce6	Photoacoustic imaging	Nanomaterials‐induced oxidative stress	^148^
	Cyanobacteria	CaAl_2_O_4_:Eu,Nd blue persistent luminescence material (PLM)	Optical imaging	Nanomaterials‐induced oxidative stress	^149^
	Cyanobacteria	Upconversion nanoparticles and Ce6	Optical imaging	Nanomaterials‐induced oxidative stress	^150^
	*E. coli*	Fe_3_O_4_@lipid nanocomposites with anti‐CD47 nanobody	Optical imaging	Immodulation	^23^
	*E. coli*	KillerRed	Optical imaging	Nanomaterials‐induced oxidative stress	^28^
	*E. coli* and *S. Typhimurium*	Glucose polymer‐ICG‐loaded silicon nanoparticles (GP‐ICG‐SiNPs)	Optical imaging	Nanocarriers for antitumor drug delivery; Immodulation	^32^
	*E. coli*	Black phosphorus nanoparticles and TRAIL	Optical imaging	Synthesis of bioactive antitumor metabolites	^109^
	*E. coli*	The l‐methionine‐*γ*‐cleaving enzyme (MdeA) and ICG	Optical imaging	Synthesis of bioactive antitumor metabolites	^27^
	*E. coli*	Gold nanoparticles and ClyA	Optical imaging	Synthesis of bioactive antitumor metabolites	^114^
	*E. coli*	TBP‐2	Optical imaging	Nanomaterials‐induced oxidative stress	^145^
	*E. coli*	TNF‐*α* decorated with gold nanoparticles	Optical imaging	Immodulation	^160^
	*E. coli*	Upconversion nanoparticles and blue‐light responsive module	Optical imaging	Immodulation	^170^
	*E. coli*	Lipid nanoparticles containing IR780, PFH, and AQ4N	Optical/Photoacoustic imaging	Nanocarriers for antitumor drug delivery	^172^
	*E. coli*	ICG and polydopamine	Photoacoustic imaging	Nanocarriers for antitumor drug delivery	^176^
	*E. coli*	CCN	Optical imaging	Synthesis of bioactive antitumor metabolites	^108^
	*Porphyromonas gingivalis*	Erythrocyte membrane	Photoacoustic imaging	Immodulation	^174^
	*S. oneidensis*	Palladium nanoparticles (Pd NPs), methylene blue (MB), and zeolitic imidazole frameworks‐90 (ZIF‐90)	Optical imaging	Nanocarriers for antitumor drug delivery	^128^
	*S. Typhimurium*	Polydopamine	Optical imaging	Nanocarriers for antitumor drug delivery	^125^
	*S. Typhimurium*	ICG‐loaded nanoparticles	Optical imaging	Nanocarriers for antitumor drug delivery	^126^
	*S. Typhimurium*	Fluorogen‐activating protein (FAP)	Optical imaging	Nanomaterials‐induced oxidative stress	^146^
	*S. Typhimurium*	Firefly‐luciferase‐expressing plasmid (*Luc‐S.T*._*ΔppGpp*_) with Ce6	Optical imaging	Nanomaterials‐induced oxidative stress	^169^
Magnetic resonance imaging‐guided nanotheranostic agent	*E. coli*	Magnetic nanoparticles	Optical imaging	Nanomaterials‐induced oxidative stress	^187^
	*Magnetospirillum gryphiswaldense*	Mn	Magnetic resonance imaging	Nanocarriers for antitumor drug delivery	^182^
	*Magnetospirillum magneticum*	Magnetite (Fe_3_O_4_) or greigite (Fe_3_S_4_)	Magnetic resonance imaging	Nanocarriers for antitumor drug delivery	^181^
	*Magnetospirillum magneticum*	Gold nanoparticles	Magnetic resonance /Photoacoustic imaging	Nanomaterials‐induced oxidative stress	^183^
	*Magnetospirillum magneticum*	Ce6	Optical imaging	Nanomaterials‐induced oxidative stress	^184^
	*Spirulina microalgae*	Pd@Au nanoparticles, doxorubicin, and Fe_3_O_4_	Optical imaging	Nanocarriers for antitumor drug delivery	^186^
PET/CT imaging‐guided nanotheranostic agent	*C. novyi*‐NT	NaGdF_4_/Tb/Ce@NaGdF_4_	CT imaging	Nanomaterials‐induced oxidative stress	^193^
	*C. novyi*‐NT	Branched gold nanoparticles (BGNP)	CT imaging	Bacterial encapsulation	^194^
	*E. coli*	^64^Cu and ^67^Cu	PET imaging	Synthesis of bioactive antitumor metabolites	^191^
	*S. Typhimurium*	Copper sulfide nanomaterials (CuS NMs) and NLG919‐GSH responsive nanoparticles	Three‐dimensional nanocomputed tomography (3D nano‐CT) imaging	Immodulation	^155^
	*Shewanella algae*	Gold nanoparticles and tetrodotoxin	CT imaging	Synthesis of bioactive antitumor metabolites	^115^

### 4.1 Assistance in therapeutic efficacy enhancement

#### 4.1.1 Bacterial encapsulation

Several factors, including insufficient target colonization, processing stress, and gastrointestinal (GI) mucosal barrier, contribute to the reduction of bacterial survival and viability during intravenous or oral administration.^91^, ^92^, ^93^ Bacterial encapsulation presents appreciable potential for clinical applications of targeted bacterial delivery based on incorporating nanoencapsulation technology with oncolytic bacteria.^94^

**FIGURE 3:**
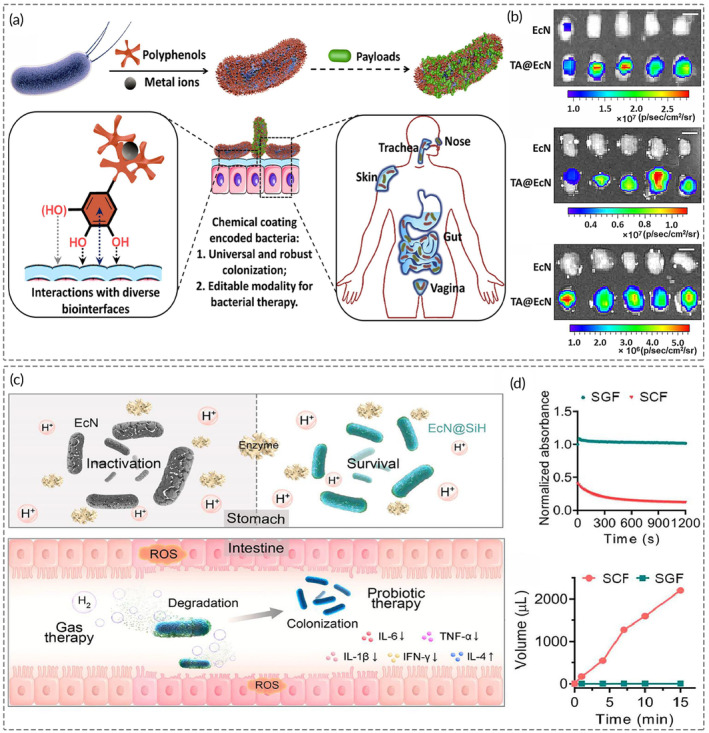
Bacterial encapsulation with nanomaterials facilitates precise colonization and environmental stress tolerance. (a) Preparation schematic of TA@EcN through supramolecular complexation with Fe^III^. (b) In vivo imaging system (IVIS) images and corresponding fluorescence intensities of the jejunum, cecum, and colon (from top to bottom) observing exposure to equivalent native EcN or TA@EcN. (c) Diagram illustrating the encapsulation/degradation process of EcN@SiH in the stomach and intestine. (d) The time‐dependent degradation kinetics and generation of H_2_ in SiH@TPGS‐PEI‐suspended SGF and SCF. Figures (a) and (b) were reproduced from ref. 25 with permission from Elsevier, Copyright 2023; figures (c) and (d) were reproduced from ref. 103 with permission from American Chemical Society, Copyright 2023.

Oncolytic bacteria may exhibit their well‐tried therapeutic effects by transient colonization of the host's GI tract mucosal surface. Thus, selecting nanomaterials with mucoadhesive properties is a staple consideration in prolonging the temporary retention of oncolytic bacteria in the GI tract.^95^ Silk fibroin is an anti‐inflammatory protein that targets ulcers and damaged areas of the intestine while generating protective nanoshells by self‐assembling on the surface of nanoparticles (from a random coil to *β*‐sheet conformation).^96^ With these ascendant traits, silk fibroin nanocoated *E. coli* Nissle 1917 (EcN) outperformed uncoated germs in a 5.8‐fold higher intestinal colonization and improved oral bioavailability. By tannic acid (TA) and Fe^III^ chelation, Luo et al. synthesized a polyphenol‐metal nanocoated EcN (TA@EcN) capable of encoding bacterial colonization and therapeutic modalities (Figure 3a).^25^ The TA coating resulted in an astounding 41.3‐fold, 39.6‐fold, and 30.1‐fold increase of EcN to adherently colonize the jejunum, cecum, and colon (Figure 3b). In the in vivo assessment, the oral bioavailability of TA@EcN was 32.7 times higher than native EcN, suggesting that nanocoatings can endow robust bacterial localization to enhance therapeutic effects synergistically.

From bacterial culture to GI tract colonization, the viability of oncolytic bacteria is paramount during manufacturing. The carboxymethyl cellulose‐chitosan (CMC‐Cht) hybrid micro‐ and macroparticles are potent, heat‐resistant, and insensitive to the potential of hydrogen (pH) changes. Singh et al. successfully encapsulated *Lactobacillus rhamnosus* GG in CMC‐Cht particles and validated the acceptable viability in all CMC‐Cht systems.^97^ The average entrapment/encapsulation efficiencies were 64% for physical encapsulation and 56% for chemically cross‐linking. Traditional oral biotherapeutics encountered inevitable manufacturing obstacles to bacterial viability, such as oxygen exposure and cellular stress reactions.^92^, ^98^ Fan et al. successfully generated metal‐phenolic networks (MPNs) on *E. coli* and *Bacteroides thetaiotaomicron* using Fe(III) ions and polyphenols.^99^ This biocompatible coating with better self‐assembly can protect bacteria from production stresses like oxygen exposure and lyophilization and can be quickly disassembled under acidic conditions. Likewise, scientists evidenced a correlation between the viability of *Lactobacillus plantarum* (*L. plantarum*) and the nanocellulose proportion in cryoprotective agents.^95^ *L. plantarum* showed the best resistance and viability after freeze‐drying at concentrations of skim milk 13.75%, trehalose 20.5%, and nanocellulose 13.75%.

Given the challenges rendered by environmental factors such as gastric acids, proteolytic enzymes, and bile salts, the NAOB hybrid should be competent to maintain metabolic activity in the harsh GI tract.^93^ Microencapsulation creates a shielding barrier between bacteria and the surrounding environment by being embedded inside or coated with nanomaterials.^100^ *Pediococcus pentosaceus* Li05 encapsulated in the microgel doped with magnesium oxide nanoparticles (MgO NPs) demonstrated better stability than in the microgel alone. It might be attributed to the filling ability of MgO NPs, which partially isolated oxygen and impeded the diffusion of hydrogen ions, bile salts, or digestive enzymes into the alginate‐gelatin microgel.^101^ Hydrogen‐bonded silicene (H‐silicene) nanosheets show excellent stability in acidic conditions, making them ideal for encapsulation.^102^ Zhu et al. reported a copolymer‐modified two‐dimensional H‐silicene nanomaterial (SiH@TPGS‐PEI) that shielded EcN from the strongly acidic environment and enzymatic lysis. Si‐H bonds are relatively active in alkaline environments but stable under acidic conditions, which made SiH@TPGS‐PEI present high stability in the simulated gastric fluid (pH 1.5) while rapidly degrading when suspended in the simulated intestinal fluid (pH 7.8) (Figure 3c,d). As expected, EcN@SiH exhibited more than 100‐fold higher viability than naked EcN after exposure to simulated gastric fluid for 15, 30, and 60 min.^103^ Similarly, the relative survival of *Salmonella* at pH 3.0 improved from 10% to 58% by encapsulation in nanoparticles composed of cationic polymers.^104^

Nevertheless, it is noteworthy that nanoparticles used as static bacterial modifications lack the capability for in situ regulation.^105^ Surface bacterial nanocoatings may also disrupt biological activities, such as membrane protein expression and flagellar rotation.^25^ Therefore, further investigation is required to determine the clinical application of encapsulation.

#### 4.1.2 Synthesis of bioactive antitumor metabolites

Bacterial‐directed enzyme prodrug therapy is an emerging cancer treatment whereby oncolytic bacteria employ enzymes to convert inactive prodrugs into antitumor substances.^106^ As a bioactive anti‐tumor molecule, the dual relationship between nitric oxide (NO) and tumors is that an insufficient concentration of NO fosters tumor growth, while a relatively high level of NO exerts antitumor effects.^107^ Zheng et al. constructed a nanobiosystem by linking carbon‐dot doped carbon nitride (CCN) with *E. coli* carrying nitric oxide synthase (NOS).^108^ Upon reaching the tumor site, photoelectrons excited from CCN headed to NO synthases within *E. coli*. This mechanism facilitated the reduction of endogenous NO_3_^−^ to cytotoxic NO, thereby instigating apoptosis in tumor cells. Chen et al. conducted a parallel investigation that combined *E. coli* with black phosphorus nanoparticles (BP NPs).^109^ When irradiated with a 635‐nm laser, *E. coli* effectively captured photoelectrons generated by BP NPs and triggered the metabolism of nitrate reduction to NO. In addition to generating endogenous cytotoxic metabolites, the nanobiohybrid can also metabolize macronutrients in the TME, like amino acids and lactate. Researchers modified *E. coli* to express the l‐methionine‐*γ*‐cleaving enzyme (MdeA) and loaded it with indocyanine green (ICG). In response to near‐infrared laser irradiation, the complex released MdeA and depleted the essential amino acid methionine (Met) to disrupt the balance of the TME.^27^ *Eubacterium hallii* deposited by iron‐polyphenol nanoparticles sustained the conversion of intratumoral lactate to butyrate, inhibiting the polarization of pro‐tumor M2‐like macrophages.^110^

Alongside catalyzing the generation of antitumor substances, biological enzymes could serve as an essential physical factor in tumor drug resistance. In 2017, According to Geller et al., intratumoral Gammaproteobacteria resulted in drug resistance in patients with pancreatic ductal adenocarcinoma, rendering them less responsive to the chemotherapy drug gemcitabine.^111^ It might attribute to the expression of a specific enzyme, cytidine deaminase (CDD), in the intratumoral bacteria. Consequently, selective inhibition of drug‐resistant enzyme activity has become a prospective approach to overcome chemoresistance. Researchers bound nitrogen‐doped carbon nanospheres (N‐CSs) competitively to the active center of CDD, effectively blocking the metabolism of gemcitabine while catalyzing the production of hydroxyl radicals (•OH) to damage tumor cells.^112^

As living organisms, the ability of oncolytic bacteria to genetically construct the expression of virulence proteins makes them a potent tumor‐killing agent. ClyA is an immunogenic pore‐forming toxin that forms the transmembrane channel either by assembling a growing pore or by creating a soluble pre‐pore in the plasma membrane.^113^ Wang et al. made a thermally‐activated biohybrid through the integration of a plasmid containing a thermally sensitive promoter and gene of ClyA into the non‐pathogenic *E. coli*, leading to the controllable ClyA expression.^114^ At 45°C, the thermally sensitive promoter enabled ClyA expression, leading to pore formation in the cancer cell membranes. *Shewanella algae* (*S. algae*) is a proficient natural tetrodotoxin producer, but wild strains typically contain limited quantities of tetrodotoxin. Henceforth, researchers obtained the optically controlled material‐assisted microbial system (called Bac@Au) by biosynthesizing gold nanoparticles (AuNPs) on the surface of hypoxia‐targeted *S. algae*.^115^ Under light irradiation, photoelectrons from AuNPs were deposited on the bacterial surface and transferred to the bacterial cytoplasm. The transference finally facilitated the in situ synthesis of the antitumor agent tetrodotoxin with a 40% increase.

Substrate limitation and metabolite toxicity pose challenges in synthesizing anti‐neoplastic drugs. High doses of pre‐drug substrates may cause undesirable off‐target toxicity despite enhancing therapy effectiveness.^116^ Besides, antitumor metabolites like tetrodotoxin often take toxicity risks. Further discussions on tetrodotoxin accumulation in normal tissues and its health implications are warranted. Overall, creating NAOB hybrids to generate bioactive antitumor metabolites presents an optimistic therapeutic tactic after ensuring safety and sustainability.

#### 4.1.3 Nanocarriers for antitumor drug delivery

Chemotherapeutic agents are the cornerstone of oncologic pharmacotherapy for their capacity to eradicate tumor cells. Scientists integrated nanomaterials with FuOXP (a prodrug conjugate of 5‐FU and oxoplatin) to construct novel drug delivery nanoparticles.^117^, ^118^ When injected into mice, approximately 10% of the nanoparticles reached tumors, showing an immense leap over most other nanocarriers (with an average reach of 0.7%). However, their targeting still needs to catch up to the ideal level. Due to the innate anaerobic targeting ability and high load volume, NAOB can be harnessed as a safe and efficient system for chemotherapy.

Uthaman et al. connected *S. Typhimurium* to hyaluronic acid (HA) microbeads encapsulating docetaxel.^119^ Specific tumor cell targeting via CD44 receptors, the microbeads were enzymatically degraded by hyaluronidases and then released docetaxel, facilitating the exact delivery of nanoparticles loaded with chemotherapeutics. Likewise, Xiao et al. exploited *Bifidobacterium infantis (B. infantis)* to diminish chemical toxicity by pinpointing the explicit delivery of the adriamycin (DOX)‐loaded nanoparticles to breast tumors, achieving a fourfold increase in DOX accumulation compared to the free location.^22^ Alongside functioning as a drug nanocarrier, *B. infantis* can also run as a pre‐seeded tumor target. Researchers first introduced *B. infantis* to locate the tumor hypoxic zone, then administered *Bifidobacterium* antibody‐modified DOX nanoparticles.^120^ The specific targeting interaction between the antibody and *B. infantis* allowed the precise conveyance and distribution of the chemotherapeutic drug DOX to kill tumor cells. Chemotherapeutic agents can also impact the TME by interfering with tumor metabolism, thus boosting the therapeutic outcome of chemotherapy. In three distinct experiments, DOX, a conventional inducer of immunogenic cell death (ICD), was bound to three different formulations: EcN carrying hyaluronidase‐hybridized albumin nanoparticles, *E. coli* MG1655 electrostatically adsorbed poly (lactic‐co‐glycolic acid) nanoparticles, and *Eubacterium hallii* loaded with iron‐polyphenol nanoparticles.^110^, ^121^, ^122^ All these formulations collectively exhibited potent effects in cancer chemo‐immunotherapy by enhancing the modulation of ICD within the intratumoral immuno‐metabolic microenvironment.

Photothermal agents (PTAs) convert light energy into heat to raise the temperature of the tumor site, triggering tumor cell death and tumor thermal ablation.^123^ However, due to the poor tumor‐targeting properties of traditional PTAs, the outcome of PTT is partially affected. The leverage of oncolytic bacteria for delivering nano‐PTAs that absorb near‐infrared light is considered a prospective approach.^124^ Sun's team created a nano‐bacteria hybrid (pDA VNP) by coating *S. Typhimurium* VNP20009 with polydopamine via oxidation and self‐polymerization.^125^ With *Salmonella*'s anaerobic targeting capacity and polydopamine's photothermal effect, tumors in pDA VNP‐treated mice shrank from Day 4 and eventually disappeared without relapse or metastasis, and all mice survived for at least 90 days. YB1, a safe *S. Typhimurium* strain, has also emerged as a popular candidate for tumor treatment. Researchers conjugated ICG loaded with nanophotosensitizers onto YB1, successfully forming bacterial‐material complexes known as YB1‐INPs.^126^ After intravenous injection of YB1‐INPs and irradiation with NIR laser, the fluorescent signal of YB1‐INPs at the tumor site increased during the treatment course. At 28 days, the tumor suppression rate of YB1‐INPs was 100%. It indicated that YB1‐driven hypoxic targeting and photothermal‐assisted bioaccumulation achieve low‐dose and high‐efficiency PTT.

Another factor that influences the efficiency of PTT is the intrinsic tumor thermal tolerance. High‐temperature treatment (above 50°C) may harm normal tissues, while low temperature (42–46°C) may yield poor therapeutic effects due to the expression of heat shock proteins (Hsps).^127^ Zhang's team engineered *Shewanella oneidensis* (*S. oneidensis*) MR‐1 to create a consortium (ZIF‐90/MB) capable of amplifying PTT.^128^ They combined MR‐1 hybridized with zeolitic imidazole frameworks‐90 (ZIF‐90) encapsulated photosensitizer methylene blue (MB). Under 660 nm and 808 nm laser, MR‐1 reduced sodium tetra‐chloropalladate (Na_2_PdCl_4_) into palladium nanoparticles (Pd NPs), displaying steady photothermal ability. In the meantime, ZIF‐90/MB produced singlet oxygen (^1^O_2_) to impair mitochondrial function, inhibit ATP production, downregulate tumor HSP expression, and eventually damage approximately 70% of cancer cells.

Nanocarriers for other cytotoxic drug delivery achieved equally favorable results. The composition of *S. Typhimurium* VNP20009 and magnetic nanoparticles (Fe_3_O_4_) loaded with ferroptosis‐inducing agent sulfasalazine enhanced ferroptosis, leading to the exceeding suppression of tumor growth.^129^ *S. oneidensis* MR‐1, engineered with the electron acceptor MnO_2_, could maintain bacterial respiration by utilizing intratumoral lactate as an electron donor. This sustained catabolism of lactate contributed imperatively to tumor suppression.^130^ The nanobiohybrid potently addressed the issues of poor drug targeting and tumor thermal tolerance during drug delivery, offering a promising alternative approach to leveraging nanobiohybrid in cancer treatment.

#### 4.1.4 Nanomaterials‐induced oxidative stress

ROS are products of oxidative stress, including superoxide anion radicals (O_2_^•−^), •OH, and ^1^O_2_.^131^ Some regulatory mechanisms involving ROS have proven to suppress tumorigenesis. As the central regulating system implicating glutathione (GSH) and nicotinamide adenine dinucleotide phosphate (NADPH), ROS deploys tumor‐suppressive effects by increasing susceptibility to cell death and promoting apoptosis.^132^ In addition, excessive ROS can interact with biomolecules (DNA, proteins, lipids, etc.), destroy their structures, affect their functions, and ultimately cause cancer cell death.^131^ Inspired by ROS's acute tumor eradication capacity, chemodynamic therapy (CDT) and PDT propose revolutionary approaches to generate high ROS in a tumor‐specific manner.^133^, ^134^

CDT capitalizes Fenton or Fenton‐like reactions to stimulate internal ROS production from endogenous sources, such as a particular concentration of mitochondrial oxygen or the continuous generation of H_2_O_2_.^135^, ^136^ However, the hypoxic microenvironment and endogenous H_2_O_2_ are deficient in achieving optimal CDT efficacy, indicating the urgency for boosting intracellular H_2_O_2_ levels.^137^, ^138^ *E. coli* was designed to overexpress NDH‐2 (respiratory streptokinase II), which accepted electrons from NADH (nicotinamide adenine dinucleotide) and transferred them to oxygen for H_2_O_2_ production.^139^ Subsequently, magnetic Fe_3_O_4_ nanoparticles (MNPs) were surface‐modified onto the bacteria to create a bioreactor as Ec‐pE@MNP. With continuous respiration, Ec‐pE@MNP consistently generated H_2_O_2_ and converted them into cytotoxic •OH by the Fenton‐like reaction, inducing severe tumor cell apoptosis and realizing a self‐supplied CDT. The overexpression of GSH in the TME, which can provoke ROS depletion, is another determinant factor impacting the effectiveness of CDT.^140^ To improve ROS generation, researchers decorated Au@Pt compounds on the surface of *E. coli*, constituting a bacterium‐based nanozyme (Bac‐Au@Pt).^141^ Bac‐Au@Pt has been validated to yield ROS at pH 6.4 efficiently. Simultaneously, IFN‐*γ* released by T cells disrupted the antioxidant defense of GSH, fostering ROS‐induced plasma membrane oxidation and apoptosis in tumor cells. *E. coli*/MnOx‐based nanospindles (EM NSs) were engineered to release DOX and Mn^2+^ ions decomposed by GSH.^142^ DOX is a potent chemotherapeutic agent, whereas Mn^2+^ ions catalyze the Fenton‐like reaction to produce •OH. EM NSs presented high safety and efficiency in tumor eradication by GSH consumption and ROS generation in chemo‐chemodynamic cancer therapy.

PDT is an emerging method for tumor treatment that offers low toxicity, minimal invasiveness, high selectivity, and easy synergism.^133^ There are two types of PDT based on ROS generation mechanisms. The type I pathway necessitates the photosensitizer absorbing light energy and generating cytotoxic ROS through electron transfer. Upon absorption of light energy, the type II photosensitizer undergoes an electron spin exchange with ground‐state oxygen (^3^O_2_), converting ^3^O_2_ to ^1^O_2_. The ^1^O_2_ subsequently oxidizes biomacromolecules and stimulates an oxidative stress response in the target cells.^133^, ^143^

The type I pathway is inherently oxygen‐independent and can efficiently produce considerable ROS even under severe hypoxia conditions (2% oxygen), but the penetrating depth of conventional lasers is often restricted.^144^ Researchers deposited aggregation‐induced emission photosensitizers on the outer membrane of *E. coli* (termed AE).^145^ AE productively generated •OH via the type I photodynamic reaction while allowing successful targeting of hypoxic orthotopic colon tumor regions. This hybrid system guided by interventional light proficiently facilitated hypoxia‐resistant PDT treatment by overcoming the limitations of light penetration depth. Moreover, engineering photosensitive bacteria through biosynthesis is another widely investigated direction to elevate the effectiveness of PDT. Guo et al. developed a genetically engineered strain of *S. Typhimurium* expressing fluorogen‐activating proteins (FAP dL5**).^146^ When employing the fluorogen (MG‐2I), the amalgamation of dL5**‐MG‐2I proved to be a robust photosensitizer, expediting the generation of phototoxic ROS to eliminate adjacent cancer cells and over‐accumulating bacteria. Type II PDT relies heavily on oxygen concentration, and the rapid oxygen consumption leads to deficient ^1^O_2_ production, making it challenging to attain the desirable therapeutic effect.^147^ A promising approach involves modifying cyanobacteria with the photosensitizer chlorin e6 (Ce6) to form the special Ce6‐integrated photosensitive cells.^148^ Under 660 nm laser irradiation, cyanobacteria's intense photosynthetic oxygenation properties continuously produced oxygen, which was immediately applied for photosensitizer integration to facilitate ^1^O_2_ synthesis. Likewise, the following two independent tests validated that the self‐oxygenating PDT system, co‐constructed with cyanobacteria and photosensitizers, required no long‐term external excitation for constant oxygenation and ^1^O_2_ supply.^149^, ^150^

#### 4.1.5 Immunomodulation

Cancer immunotherapy leverages the patient's immune system to initiate and maintain the inherent defensive mechanisms intended to control and eliminate tumors.^151^, ^152^ There are three dominant strategies for NAOB‐based cancer immunotherapy. One entails making immune‐related cells redistribution and reactivation to recognize and attack tumor cells. The second approach is to express and secrete cytokines that target specific parts of the immune system. Additionally, oncolytic bacteria can serve as immunological adjuvants to collaborate combination therapy with immune checkpoint blockade (ICB).

Numerous immune cells, such as macrophages and dendritic cells (DCs), are immunosuppressed or hypo‐immunoreactive in the tumor immunosuppressive microenvironment.^153^, ^154^ Researchers biosynthesized copper sulfide nanomaterials (CuS NMs) within *S. Typhimurium* VNP20009 to create the nanosystem _CuS_VNP20009_NB_, aiming to target and repolarize tumor‐associated macrophages (TAMs).^155^ TAM constitutes a pivotal factor correlated with immune resistance, while LPS in VNP20009 is a well‐accepted agent for TAM repolarization.^156^ Experimental findings indicated that _CuS_VNP20009_NB_ could accumulate in tumor tissues and reprogram immunosuppressive M2‐type macrophages into immunostimulatory M1‐type macrophages to boost the immune response. The tumor cell lysate‐coated polydopamine nanoparticles (PDA@CL) were designed to transfer tumor‐associated antigens (TAAs) precisely, alleviating the restricted activation of CD8 T cells in the TME due to insufficient antigen presentation by DCs.^157^ Then, engineered *Salmonella* (EnS) was encapsulated in PDA@CL (EnS@PDA@CL).^158^ In animal experiments, the quantities of DCs and CD8 T cells in the EnS@PDA@CL group were almost three times higher than in the PBS group, verifying that EnS@PDA@CL intensively promoted antigen cross‐presentation and subsequent CD8 T cell stimulation by DCs.

**FIGURE 4:**
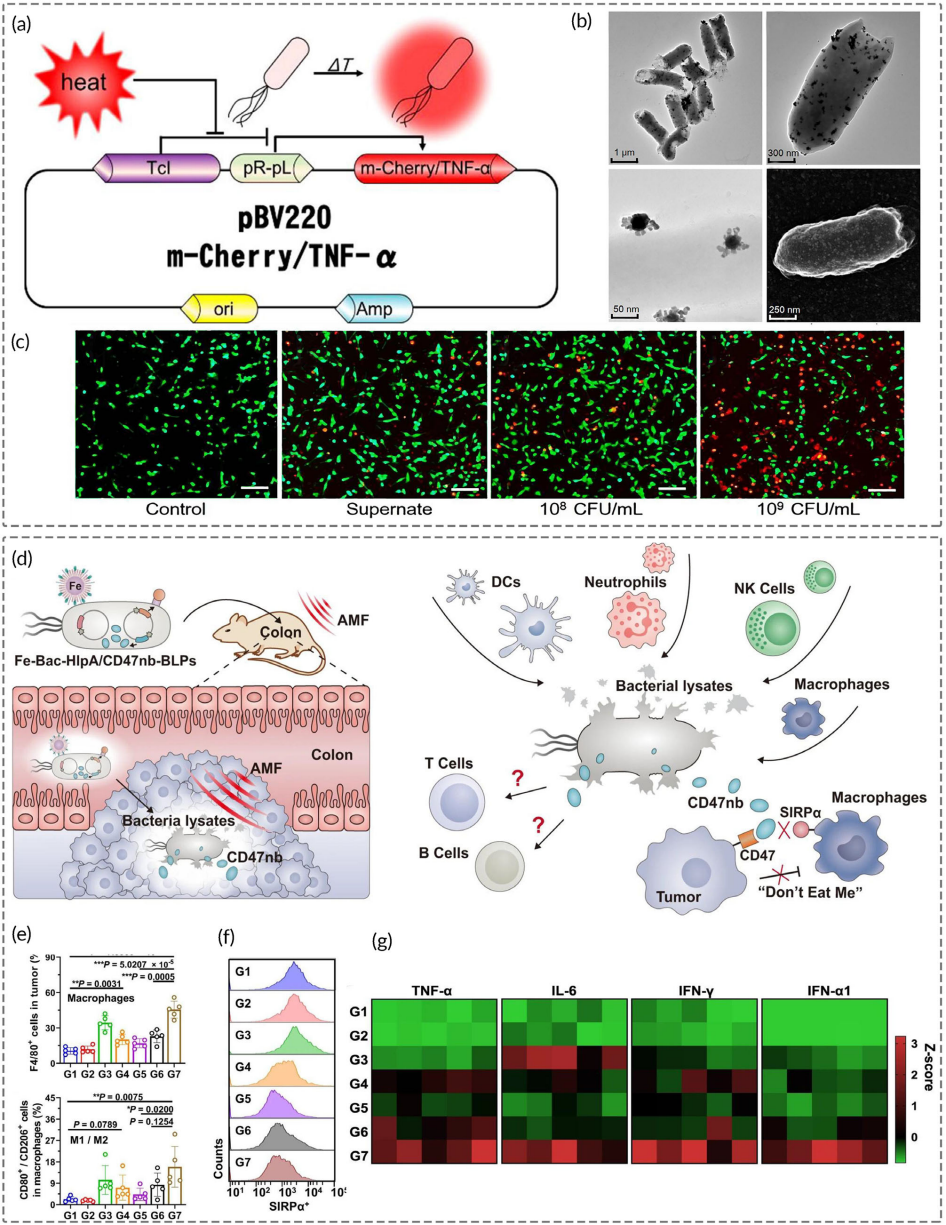
NAOB enhances the effectiveness of tumor immunotherapy by reactivating and sustaining the patient's immune system. (a) Noninvasive bacterium *Escherichia coli* MG1655 expressed m‐Cherry/TNF‐*α*. (b) TEM and SEM micrographs of thermally sensitive programmable bacteria (TPB) and surrounded AuNPs. (c) Fluorescence live/dead cell images of 4T1 cells with a blank control treatment were taken. (d) Potential mechanism of immune responses triggered by CD47nb pre‐expressed within the *E. coli*. (e) The altered quantity/phenotype of macrophages and (f) the expression level of SIRP*α* on TAMs validated the possible oncolytic mechanism of CD47nb, which relies on the CD47‐SIRP*α* signaling pathway to induce phagocytosis of tumor cells. (g) Cytokine concentrations of indicated groups showed the activation of type I IFN signaling pathway by AMF‐Bac. Figures (a)–(c) were reproduced from ref. 160 with permission from American Chemical Society, Copyright 2018; figures (d)–(g) were reproduced from ref. 23 with permission from Springer Nature, Copyright 2023. NAOB, nanomaterial‐assisted oncolytic bacteria; SEM, scanning electron microscopy; TEM, transmission electron microscopy.

Cytokines play a paramount regulatory role in cell development, differentiation, growth, and survival.^159^ Scientists constructed a thermosensitive drug delivery system in which programmable *E. coli* MG1655, expressing TNF‐*α*, was decorated with bio‐mineralized AuNPs.^160^ When exposed to NIR light, the AuNPs induced *E. coli* to precisely regulate the expression of TNF‐*α* through controlled heat generation (Figure 4a–c). Cytotoxic protein presentation led to apoptotic cell death, underscoring the potency of bacteria‐based antitumor cytokines delivery. Wang and colleagues devised a nano‐STING agonist‐decorated microrobot incorporating *S. Typhimurium* VNP20009 and mitochondria‐targeted NPs.^161^ The sophisticated microrobot enabled the concurrent delivery of the STING agonist 2′3′‐cyclic guanosine monophosphate‐adenosine monophosphate (cGAMP) and releasement of mtDNA via oxidative stress. Exogenous cGAMP and endogenous mtDNA demonstrated a synergistic augmentation of cyclic guanosine monophosphate‐adenosine monophosphate synthase (cGAS)/STING signaling, which dramatically bolstered the immunotherapeutic success of STING agonists. Nano‐bacterial complex expressing tumor necrosis factor‐associated apoptosis‐inducing ligand (TRAIL) resembled assisting tumor apoptosis, activating T‐lymphocytes, and releasing proinflammatory cytokines.^109^

Over the past decade, there have been significant advances in cancer immunotherapies, with immune checkpoint inhibitors as a prospective tool for activating self‐immunity against tumors.^162^ The nanobiohybrid synergized with prevalent ICBs, including the anti‐programmed death protein 1 (PD‐1) and PD‐L1 proteins, have been validated to potentiate the immune response by blocking the T‐cell inhibitory pathway and promoting effector T‐cell activation.^163^, ^164^ CD47 is expressed in human cells but significantly upregulated in nearly all tumor cells. Through interacting with macrophages, CD47 transmits the “don't eat me” signal to inhibit macrophage phagocytosis and facilitate tumor immune evasion.^165^ CD47 has emerged as a compelling immunological target alongside PD‐1/PD‐L1. Tal Danino's team constructed a synchronized lysis circuit (eSLC) in *E. coli* that colonized tumors to cleave and release the encoded nanobody antagonist of CD47 (CD47nb).^31^ In BALB/c mice with tumors on both sides, intratumoral administration of eSLC‐CD47nb resulted in rapid and sustained clearance of tumor cells within approximately 10 days of treatment initiation. Furthermore, researchers utilized alternating magnetic fields (AMFs) to manipulate tumor‐homing *E. coli*. Fe_3_O_4_@lipid nanocomposites enabled controlled motion via the magnetic field, which enhanced tumor targeting and facilitated the release of CD47nb pre‐expressed within the bacteria.^23^ This treatment exhibited remarkable therapeutic efficacy in both in situ and distal colon tumor models in mice, leading to rapid and long‐lasting tumor clearance (Figure 4d–g).

#### 4.1.6 Others

Various studies under the International Microbiome Consortium have linked dysbiosis to metabolic syndromes, autoimmune diseases, autism, and tumors.^166^ Nanomaterials can regulate flora signaling and metabolites in the TME. As stated, intratumoral bacteria incurred gemcitabine resistance.^111^ Zhang et al. designed a nanoformulation responsive to both pH and enzymes to counteract bacterial‐induced resistance, encapsulating gemcitabine modified with HA and the antibiotic ciprofloxacin.^167^ This nanoformulation targeted and controlled drug release in the acidic and hyaluronidase‐rich TME and killed intratumoral bacteria to overcome chemotherapy resistance. It also promoted antigen‐presenting dendritic cell maturation and depleted immunosuppressive myeloid‐derived suppressor cells in bacterially infected tumors to activate T cell‐mediated immune responses. Resembly, mucin‐crosslinked antibiotics and chemotherapeutic drugs have produced outstanding outcomes in eliminating microbially induced tumor resistance.^168^ Hence, conducting nanobiohybrid to regulate microbial flora may pave the way for a groundbreaking direction in cancer therapy.

### 4.2 Assistance in the diagnosis and treatment of nanotheranostic agents

#### 4.2.1 Optical/photoacoustic imaging‐guided nanotheranostic agent

**FIGURE 5:**
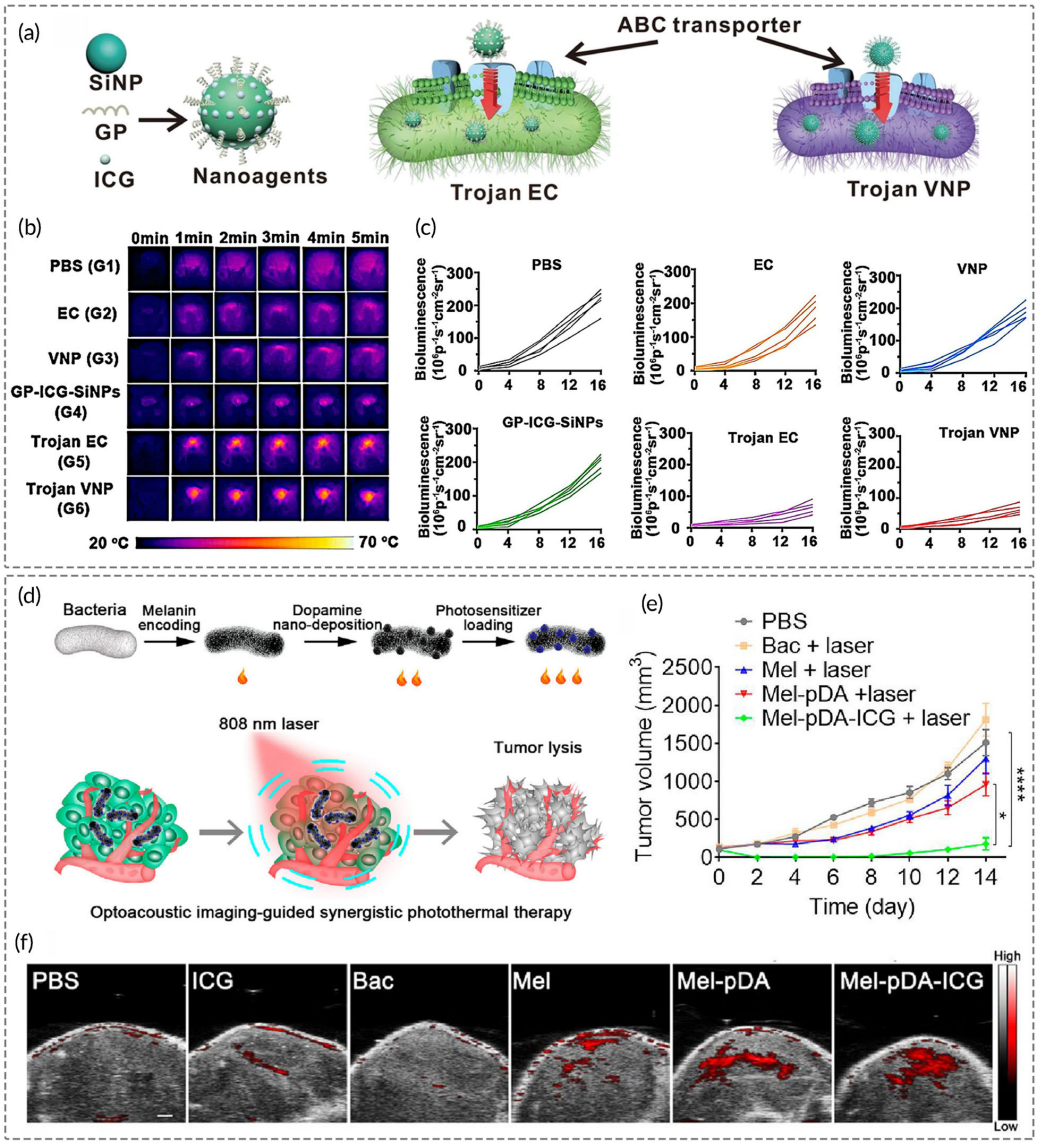
Improving diagnosis capabilities with oncolytic bacterial nanotheranostic agents for optical/photoacoustic imaging‐guided therapy. (a) Schematic graphic of the synthesis process of bacteria loaded with GP‐ICG‐SiNPs (Trojan EC). (b) Infrared images and (c) semi‐quantitative analysis of bioluminescence intensity in mouse brains with glioblastoma multiforme (GBM) treated with PBS, EC, VNP, GP‐ICG‐SiNPs, Trojan EC, and Trojan VNP under 808 nm laser irradiation. (d) Mechanism of triple photoacoustic imaging and tumor inhibition of ICG and polydopamine deposited *Escherichia coli*. (e) The tumor growth curves of each group were recorded over a 14‐day treatment period. (f) Photoacoustic tomography of tumors after 2‐h injection with equivalent volumes of PBS, ICG, bacteria (Bac), bacteria producing melanin (Mel), Mel attached with pDA (Mel‐pDA), and Mel‐pDA‐ICG. Figures (a)–(c) were reproduced from ref. 32 with permission from Springer Nature, Copyright 2022; figures (d)–(f) were reproduced from ref. 176 with permission from American Chemical Society, Copyright 2023.

Nanotheranostic agent‐mediated optical imaging, including bioluminescence and fluorescence imaging technologies, not only advances the detection of bacterial localization and proliferation in solid tumors but also augments the precision of diagnosis and treatment. Specifically, attenuated *S. Typhimurium* expressed firefly‐luciferase (*Luc‐S.T*._*ΔppGpp*_) could provide uniform imaging of entire tumors while exciting the photosensitizer Ce6 to inhibit opaque melanoma by bioluminescence‐induced PDT.^169^ NIR fluorescence imaging is a hot topic in the field of fluorescence imaging. NIR‐light responsive *E. coli* Nissle 1917, combined with the upconversion nanoparticles, are qualified to noninvasively activate the internal blue‐light‐responsive module embedded in the bacterial system and released TNF‐*α* for precise tumor ablation.^170^ Akin, *S. Typhimurium* VNP20009 aggressively internalized nanoprobes GP‐ICG‐SiNPs via the ATP‐binding cassette (ABC) transporter protein pathway, permitting NIR fluorescence imaging across the blood‐brain barrier and culminating successful glioblastoma photothermal immunotherapy (Figure 5a–c).^32^ Focused ultrasound ablation surgery (FUAS) employs concentrated ultrasound to ablate target lesions through a combined thermal‐mechanical effect.^171^ Researchers designed *E. coli* carrying encoded gas vesicles (GVs) for ultrasound imaging, leveraging the genetically engineered bacterial vector to enhance ultrasound penetration.^172^ This methodology allows the incorporation of GVs‐*E. coli* to transport lipid nanoparticles containing the chemotherapeutic drug banoxantrone dihydrochloride (AQ4N), guiding multiple imaging and chemotherapy combined with FUAS ablation.

Photoacoustic imaging (PAI) represents an emerging imaging modality aiming to detect mechanical waves from light absorption by endogenous or exogenous chromophores within the tissue.^173^ Endogenous chromophores, such as melanin, exhibit light absorption at specific wavelengths. Erythrocyte membrane‐nanocoated *Porphyromonas gingivalis* (*Pg*) served as a photoacoustic probe by promoting melanin secretion for bimodal ultrasonic/photoacoustic (US/PA) imaging‐guided photothermal immunotherapy.^174^ Various exogenous chromophores, including metal and ICG, can boost the sensitivity and precision of PAI. Zhang et al. engineered metal‐organic frameworks nanosheets based on a bacteriochlorin ligand, capable of performing PAI‐guided hypoxic tumor ablation through a synergistic mechanism of type I and II PDT.^175^ Organic dyes also possess powerful imaging properties. Guo et al. developed a photosensitizer by nano‐deposition of ICG and polydopamine from melanin‐expressing *E. coli* (Mel‐pDA‐ICG), which produced stable triple photoacoustic and photothermal effects for PAI‐guided synergistic PTT in colon and breast cancer mouse models (Figure 5d–f).^176^

#### 4.2.2 Magnetic resonance imaging‐guided nanotheranostic agent

Magnetostrophic bacteria (MTB) constitute a group of bacteria that absorb Ferrum from the external environment and synthesize membrane‐encapsulated single‐domain magnetite (Fe_3_O_4_) or greigite (Fe_3_S_4_) crystals, known as magnetosomes. MTB can function as the natural magnetic resonance imaging (MRI) contrast agent to generate positive and negative contrast while also serving as MRI‐specific marker molecules and probes with high affinity and strong visibility for various solid tumors.^177^, ^178^, ^179^, ^180^ Capitalizing on the outstanding tumor imaging and anaerobic targeting properties of MTB and magnetosomes, numerous scientists have integrated them with nanomaterials to exert synergistic antitumor effects. Bacterial magnetic nanoparticles (BMPs), biomineralized by *Magnetospirillum magneticum* and coated with a biomembrane layer, not only improved *T*_2_‐weighted MRI negative contrast at the tumor site by 25% but also instigated in vivo PTT for the ultimate eradication of tumors.^181^ In addition, manganese (Mn)‐doped magnetosomes (MagMn) provide more pronounced *T*_1_/*T*_2_ dual‐mode MRI signals.^182^ Analyzed through experiments, it was evident that the transverse relaxivity (r2) and longitudinal relaxivity (r1) of MagMn surpassed those of other contrast mediums. When modified with tumor‐targeting peptide iRGD, MagMn selectively accumulated in tumor tissue and guided MRI‐induced photothermal antitumor therapy.

Integrating magnetosomes with nanomaterial presents opportunities for multimodal imaging and collaborative therapeutic interventions. MSC‐Au, a therapeutic agent formed by AuNPs and magnetosomes, offers distinctive photothermal properties with the additional capacity to metabolize glucose into gluconic acid, triggering ROS generation upon interaction with magnetosomes. Employing MRI and PAI guidance, MSC‐Au exhibited synergistic combined CDT/PTT in diverse animal models.^183^ Wang and collaborators introduced the photosensitizer Ce6 into *Magnetospirillum magneticum* strain AMB‐1 to fabricate AMB‐1/Ce6 micromotors, expanding the potential applications of fluorescence imaging. After the injection of micromotors, AMB‐1/Ce6 achieved dual tumor‐killing effects of PDT and AMB‐1‐induced ROS damage under magnetic field guidance and laser irradiation.^184^ This trial showcases MTB to act as drug carriers and drugs in the meantime, providing an innovative and multifunctional strategy for cancer therapy.

Magnetic‐responsive bacteria are strains that undergo magnetization through the binding of magnetic materials. Evidence has shown that modification of *Spirulina microalgae* (*Sp*.) with nano‐contrast agents, such as superparamagnetic magnetite suspensions, enabled the tracing of bacterial behavior in vivo by MRI.^185^ Researchers designed a biohybrid magnetic microrobot consisting of photothermal conversion agent Pd@Au NPs, the chemotherapeutics doxorubicin, and Fe_3_O_4_ together with *Sp*.^186^ Under a rotating magnetic field, this microrobot achieved highly targeted and synergistic chemo‐PTT with optical imaging mediation. *E. coli*, with its high sensitivity, has been fond of many scientists in making non‐magnetic bacteria magnetic. A micro‐robotic system is composed after loading magnetic nanoparticles and an internal fluorescent protein DiR on the probiotic *E. coli*, which encodes the NDH‐2 enzyme.^187^ The system triggered magnetothermal ablation and NDH‐2‐induced ROS damage, effectively rendering apoptotic responses in cancer cells under fluorescence imaging.

#### 4.2.3 PET/CT imaging‐guided nanotheranostic agent

Positron emission tomography (PET) imaging, characterized by its high sensitivity, is the most prevalent tool in diagnosing and treating solid tumors. Live bacteria have proven to be successful carriers for delivering radionuclides. For instance, fluorescent dye Cy5.5 or ^125^I labeled attenuated *S. Typhimurium* achieved fluorescent and nuclear dual‐modality imaging of breast cancer and colon cancer.^188^ ^18^F‐fluorodeoxysorbitol (FDS) PET was capable of imaging *E. coli* and visualizing the colonization and proliferation of tumor‐targeting Gram‐negative bacteria in mouse tumor models.^189^ Targeted radionuclide therapy (TRT) is a rapidly growing field that utilizes substances like radiolabeled molecules, radioisotopes, nanoparticles, or microparticles to target cancer cells with cytotoxic *α* and *β* particles.^190^ An integrated microbe‐based pretargeting approach used a bacteria‐specific radiopharmaceutical to target solid tumors, employing *E. coli* Nissle 1917 as a delivery vehicle.^191^ This approach utilizes the siderophore‐mediated metal uptake pathway to selectively concentrate ^64^Cu and ^67^Cu in complex with yersiniabactin (YbT) within transgenic bacteria. PET imaging with ^64^Cu‐YbT enabled visualization of bacteria within the tumor, while ^67^Cu‐YbT delivered a cytotoxic dose to the surrounding cancer cells. Cancerous mice carrying MC38 and 4T1 tumors showed impressive tumor reduction and prolonged survival with ^67^Cu‐YbT.

Computed tomography (CT) is a commonly utilized technique in contemporary clinical imaging due to its cost‐effectiveness, quick examination durations, and user‐friendly operation.^192^ Researchers developed a nano‐bio emulsion that collaborated X‐ray PDT with oncolytic bacterial therapy. The emulsion contained photosensitizer‐coated nanoscintillators (NaGdF_4_/Tb/Ce@NaGdF_4_) and *C. novyi*‐NT spores.^193^ Results indicated that image‐guided X‐ray PDT expressed elevated levels of apoptotic cell death in cancerous tissue observed under CT imaging. An alternative method in tumor therapy involves applying branched gold nanoparticles (BGNPs) coating on *C. novyi*‐NT spores for CT‐guided precise tumor therapy.^194^ Leveraging the anaerobic targeting specificity and tumor oncolytic capabilities of spores, guided by CT imaging, the deployment of this composite spore manifested robust antitumor effects in the PC3 prostate‐tumor‐bearing mouse model.

Other alternative forms of modalities find application in the realm of cancer treatment. Zhang and colleagues have detailed an integrated nanosystem (Bac@BNP) comprising *E. coli* and bismuth sulfide nanoparticles (BNPs), demonstrating its capacity to augment radiotherapy sensitivity.^30^ When exposed to X‐ray irradiation, the synergistic effect of bacterial ClyA and BNPs, possessing high‐Z element radiosensitivity, induced a substantial production of ROS and relevant DNA damage.

Bacteria‐specific molecular imaging is poised to expand the indications for bacterial‐mediated tumor therapies and facilitate the clinical applicability of precision medicine. Optical/photoacoustic imaging‐guided nanotheranostic agents mitigate pigment interference, enhancing signal aggregation and photoacoustic signal generation at specific sites. MRI, leveraged for high spatial resolution and absence of radiation, confronts limitations due to magnetic pores and contrast agents addressed effectively by MTB and magnetic‐responsive strains.^195^ PET/CT achieves remarkable sensitivity (10^−10^ to 10^−12^ m), enabling bacteria‐tumor co‐localization, quantitative assessment, and accurate TRI diagnosis.^170^ Nevertheless, only a few studies have employed oncolytic bacteria as imaging and medical probes, and further experimental validation is warranted.

## 5 POTENTIALITIES OF NAOB: CONSTRAINTS AND OPPORTUNITIES

Several issues necessitate further investigation when utilizing the NAOB hybrid in solid tumor diagnosis. The metabolism and motility of nanobacteria can directly affect diagnostic results and imaging quality. Compared with antibody‐mediated AuNP surface modification on *C. difficile* spores, the non‐specific modification of AuNPs on the *Bifidobacterium breve* led to lower tumor enrichment. This decrease can be attributed to the dilution resulting from bacterial cell growth and division.^196^ Bacterial motility may induce artifacts or blur images, affecting their localization and morphology accuracy.^197^ Moreover, various imaging modalities encounter challenges when utilizing NAOB. Optical imaging is limited by its penetration depth (1 cm) and usually receives small molecule interferences such as hemoglobin, water, and cytochromes.^198^ PET imaging tracers applied in preclinical and clinical studies may accumulate non‐specifically in normal organs or off‐target lesions.^195^

A crucial therapeutic consideration is the safety and off‐target possibility of NAOB. Many studies are now taking endogenous stimulants to detect and manipulate the nanobiohybrid. The acidity in the TME is often applied as an endogenous indicator. However, it is crucial to consider the similarly low lysosomal pH in normal cells, which may pose a risk of off‐target damage from the hybrids.^134^, ^199^ Additionally, nanoparticles, like gold and silver, are complex to degrade and can have adverse health effects if internally deposited. Green biomaterials are a novel category of biodegradability, biocompatibility, nontoxicity, and swift removal characteristics.^200^ Studies have shown that multifunctional and nontoxic green graphene emerges as a promising candidate in cancer nanodiagnostics, and microbial‐mediated synthesis of eco‐friendly metal nanoparticles exhibits remarkable efficacy in cancer therapy.^201^, ^202^ Therefore, it is necessary to optimize the performance of oncolytic bacteria and green biomaterials by operating different tactics in various nanomedicine applications.

Based on the preceding, it is imperative to conduct further research in the cooperation of oncolytic bacteria and nanomaterials. Ensuring the safety of nanomaterial‐loaded bacteria is paramount, preferably with controlled release into tumor cells under external detection and regulation. Next, we advocate for extensive clinical research to explore alternative practical methods for promoting nanobiohybrid safety, efficacy, and metabolism. Optimizing imaging techniques, improving contrast agents, and reducing background disturbances are also essential parameters for reinforcing the stability of the nanobiological system. Although nanobiohybrids still have room for improvement, the combination of both properties and advantages has shown substantial potential for development and to become innovative and intelligent systems for future solid tumor treatment.

## 6 CONCLUSION

Oncolytic bacteria‐mediated tumor therapeutics offer superiority by leveraging their motility and penetration to directly or indirectly eliminate tumor cells while concurrently augmenting the anticancer immune response. Nanomaterials and nanocomposites attract global attention from researchers due to their transcendent performance in transmission, encapsulation, and conductivity. Combining these two approaches can complement the strengths and address their respective limitations. With the continuing development of biotechnology, nanomaterial‐modified oncolytic bacteria can serve as potent tumor‐targeting transmitters, drug carriers, and immunomodulators. While some shortcomings, like safety concerns and off‐target possibility, require urgent attention, the future of utilizing NAOB in tumor therapy holds promise as a prospective methodology for clinicians and researchers aiming to broaden the applications of bacterial‐mediated tumor therapy and advance precision medicine.

## AUTHOR CONTRIBUTIONS

**Xiangdi Zeng:** Conceptualization; resources; software; writing – original draft; writing – review and editing. **Qi Chen:** Supervision; validation; visualization. **Tingtao Chen:** Conceptualization; resources; supervision; validation; visualization.

## CONFLICT OF INTEREST STATEMENT

The authors declare no conflict of interest.

### PEER REVIEW

The peer review history for this article is available at https://www.webofscience.com/api/gateway/wos/peer-review/10.1002/btm2.10672.

## Data Availability

Data sharing is not applicable to this article as no new data were created or analyzed in this study.
